# Innate Immune Responses in the Outcome of Haploidentical Hematopoietic Stem Cell Transplantation to Cure Hematologic Malignancies

**DOI:** 10.3389/fimmu.2019.02794

**Published:** 2019-11-28

**Authors:** Elisa Zaghi, Michela Calvi, Clara Di Vito, Domenico Mavilio

**Affiliations:** ^1^Unit of Clinical and Experimental Immunology, Humanitas Clinical and Research Center, Milan, Italy; ^2^Department of Medical Biotechnologies and Translational Medicine (BioMeTra), University of Milan, Milan, Italy

**Keywords:** innate lymphocytes, haploidentical hematopoietic stem cell transplantation, immune-reconstitution, natural killer cells, innate lymphoid cells, γδ T cells, alloreactivity

## Abstract

In the context of allogeneic transplant platforms, human leukocyte antigen (HLA)-haploidentical hematopoietic stem cell transplantation (haplo-HSCT) represents one of the latest and most promising curative strategies for patients affected by high-risk hematologic malignancies. Indeed, this platform ensures a suitable stem cell source immediately available for virtually any patents in need. Moreover, the establishment in recipients of a state of immunologic tolerance toward grafted hematopoietic stem cells (HSCs) remarkably improves the clinical outcome of this transplant procedure in terms of overall and disease free survival. However, the HLA-mismatch between donors and recipients has not been yet fully exploited in order to optimize the Graft vs. Leukemia effect. Furthermore, the efficacy of haplo-HSCT is currently hampered by several life-threatening side effects including the onset of Graft vs. Host Disease (GvHD) and the occurrence of opportunistic viral infections. In this context, the quality and the kinetic of the immune cell reconstitution (IR) certainly play a major role and several experimental efforts have been greatly endorsed to better understand and accelerate the post-transplant recovery of a fully competent immune system in haplo-HSCT. In particular, the IR of innate immune system is receiving a growing interest, as it recovers much earlier than T and B cells and it is able to rapidly exert protective effects against both tumor relapses, GvHD and the onset of life-threatening opportunistic infections. Herein, we review our current knowledge in regard to the kinetic and clinical impact of Natural Killer (NK), γδ and Innate lymphoid cells (ILCs) IRs in both allogeneic and haplo-HSCT. The present paper also provides an overview of those new therapeutic strategies currently being implemented to boost the alloreactivity of the above-mentioned innate immune effectors in order to ameliorate the prognosis of patients affected by hematologic malignancies and undergone transplant procedures.

## Introduction

Allogeneic (allo-) hematopoietic stem cell transplantation (HSCT) represents the best curative approach for patients affected by high-risk hematologic malignancies and several genetic disorders (1). In the absence of human leukocyte antigen (HLA)-identical siblings, HLA-haploidentical (haplo) related donors are a source of hematopoietic stem cells (HSCs) immediately available for almost any patients in need (2).

However, the first developed protocols of haplo-HSCT were mainly associated with graft rejection, high degree of treatment-related mortality (TRM) and severe graft-vs.-host-disease (GvHD) due to the partial HLA-mismatch between donors and recipients. This poor clinical outcome was also worsened by the increased risk of developing opportunistic infections, a phenomenon associated with a delayed immune-reconstitution (IR) following the transplant. On the other hand, HLA-mismatch remarkably boosted the so-called Graft-vs.-Leukemia (GvL) effect that eradicates those malignant cells surviving conditioning regimes (3, 4). Hence, the mechanisms inducing both GvHD and GvL rely on immunologic alloreactivity that, indeed, represents the bad and good side of the same coin in both allogeneic and haplo-HSCT. The possibility to improve GvL while limiting life-threatening side effects have firmly driven the development of new clinical protocols of haplo-HSCT delivering better clinical outcomes. In this context, a better understanding of both kinetics and mechanisms of IR is key to improve the prognosis of patients undergone haplo-HSCT and limit its side effects (5–13).

Several lines of evidences clearly showed that a full recovery of adaptive immune responses in transplanted patients take long time. Indeed, adaptive B- and T-cell effector-functions are either lacking or not completely competent for several months after haplo-HSCT, thus leaving the patients in a deadly condition of immune-deficiency. On the other hand, innate immune cells reconstitute early after haplo-HSCT, thus ensuring a certain degree of immune-protections in the first days/weeks after the transplant (3, 14). In particular, neutrophils and monocytes recirculate at levels similar to those of healthy individuals 1 month after the infusion of HSCs, while innate lymphocyte IR starts from the 2^nd^ week after the transplant (15–17). Nonetheless, quite a few cell compartments of innate immunity are greatly impaired in their functions early after haplo-HSCT (18, 19). This scenario enforced the implementation of graft manipulations in allo-and haplo-HSCT setting (i.e., αβ T and/or B cell depletion) able to preserve Natural Killer (NK), gamma-delta (γδ) T and innate lymphoid (ILCs), thus avoiding a prolonged immune suppression and speeding their IR early after the transplant (Table 1) (12, 26–28). In particular, NK and γδ T cells have been shown to recover faster in those recipients receiving αβ T cell depleted grafts rather than the conventional CD34^pos^ conventional counterparts in the context of the haplo-HSCT setting (25, 28, 29).

**Table 1:** Main results of different haplo-HSCT protocols with relative clinical outcomes and immunological recovery.

**Sample size and disease**	**haplo-HSCT platform**	**Conditioning**	**Relapse/NRM**	**aGvHD/cGvHD**	**Clinical outcomes**	**Immune-reconstitution findings**	**References**
67 AML 37 ALL	G-CSF and TCD using CD34+ cell immunoselection	TBI Thiotepa Fludarabine ATG	Engraftment: 99% Relapse: 13,6% NRM: 36.5%	aGvHD: 8% cGvHD: 7.1%	EFS rate for AML: 48% ± 8% EFS rate for ALL: 46% ± 10%	CD4+ T cell count: from 100 ± 40/mm^3^ to 200 ± 20/mm^3^ for 10 months; CD8+ T cell count: 230 ± 80/mm^3^ day +60; 570 ± 80/mm^3^ on day +300; CD16+ NK cell count: 400/mm^3^ stably by day +30	(5)
66 ALL 51 AML 47 CML 7 MDS	NMAC TCRep	ATG CsA (d −9) MMF (d −9 to +30) Methotrexate (d +1, +3, +6, +11)	Probability of relapse: 12% at 2 years for standard-risk Probability of relapse: 39% at 2 years for high-risk	aGvHD (III–IV): 23% cGvHD: 47%	DFS: 68% at 2 years for standard-risk DFS: 42% at 2 years for high-risk	Neutrophil counts recover between 13 and 14 days; quick recovery of NK cells; CD8+ T-cell recovery starts at 2^nd^ month after the transplant; B- cell reconstitution starts at 6th month; CD4+ T-cell recovery is slower and can require till 1 year	(20, 21)
67 hematologic malignancies 1 paroxysmal nocturnal hemoglobinuria	NMAC TCRep	Cy (d −6, −5, +3, or +3/+4), fludarabine (d −6 to −2) TBI (d −1) tacrolimus MMF	Probabilities of relapse:51% at 1 year NRM: 4% at days 100; 15% at 1 year Graft failure: 13%	aGVHD (II-IV): 34% at day 200 aGVHD (III-IV): 6% at day 200	OS: 46% at 1 year; 36% at 2 years EFS: 34% at 1 year; 26% at 2 years	The median times to neutrophil recovery (>500/μL): day +15; The median times to platelet recovery (>20,000/μL): day +24	(22)
52 AML 16 ALL 15 MDS	Unmanipulated G-CSF mobilized PB with *in vivo* TCD MAC or RIC		NRM: 14% (MAC) or 9% (RIC) at 3 years Incidence of relapse: 44% (MAC) or 58% (RIC) at 3 years	aGVHD (II-IV): 16% (MAC) or 19% (RIC) cGvHD: 30%(MAC) or 34% (RIC) at 3 years	OS: 45% (MAC) or 46% (RIC)	Platelet count: 20,000/ul at 17 days; NK count: >100/ul from 3 months; CD8 count: >200/ul from 3 months; CD4+ count: >200/ul from at 1 year	(23)
57 AML 14 ALL CML 1 MM 8 HL 4 MDS 2 MFI NHL 1 Plasma Cell Leukemia	G-CSF primed, unmanipulated BM MAC = 68 or RIC = 29	TBF ATG Methotrexate CsA MMF basiliximab	TRM: 36 ± 65% (MAC) or 28 ± 9% (RIC) relapse: 22 ± 6% (MAC) or 45 ± 11% (RIC)	100 day Cumulative Incidence of aGvHD (II-IV): 31 ± 5% Cumulative Incidence of overall cGvHD: 12 ± 4% at 2 years	OS: 48 ± 7% (MAC) or 29 ± 10% (RIC) DFS: 43 ± 7% (MAC) or 26 ± 10% (RIC)	100 day Cumulative Incidence of neutrophil engraftment: 94 ± 3% 100 day Cumulative Incidence of platelet engraftment: 84 ± 4%	(24)
80 acute leukemia (AL) in pediatric children	Negative depletion of αβ T and B cells MAC	ATG (d −3, −5)	2 graft failure Relapse: 24% NRM: 5%	aGVHD (I/II): 30% cGVHD-free survival: 71% at 5 years	DFS: 71,4% (ALL) or 67.5% (AML)	CD3+ T cells/μL: 231 (1–1,618); CD4+ T cells/μL: 19 (0–442) and CD8+ T cells/μL: 24 (0–910) γδ T cells/μL: 181 (1–1,335) CD3-CD56+ NK cells/μL: 236 (47–1,813) CD19+ B cells/μL: 0 (0–20)	Clinical trial: NCT01810120 (25)

*aGvHD, acute Graft vs. host disease; ALL, Acute lymphoid Leukemia; AML, Acute myeloid Leukemia; ATG, Anti-thymocyte globulin; BM, Bone marrow; CsA, Cyclosporine A; cGvHD, chronic Graft vs. host disease; CML, Chronic myeloid Leukemia; Cy, Cyclophosphamide; d, days; DFS, Disease free survival; EFS, Event free survival; G-CSF, Growth colony stimulating factor; HL, Hodgkin lymphoma; MAC, Myeloablative conditioning; MDS, Myelodisplastic syndrome; MFI, Myelofibrosis; NHL, Non-Hodgkin lymphoma; MM, multiple myeloma; MMF, Mycophenolate mofetil; NMAC, Non-myeloablative conditioning; NMR, Non relapse mortality; OS, Overall survival; PB, Peripheral blood; RIC, Reduced intensity conditioning; TBI, Total body irradiation; TBF, Thiotepa, Busilvex, Fludarabine; TCD, T cell depletion; TCRep, T cell repletion. In immune-reconstitution findings column the cell counts are defined as mean ± SD (first row) or mean (range) at 1 month (last row)*.

### Graft vs. Host Diseases and Opportunistic Infections

One of the main complications affecting the positive clinical outcomes of allo-HSCT is still represented by the donor-derived alloreactive T cell responses against host tissues, a phenomenon inducing the onset of GvHD mainly affecting skin, gastrointestinal tract and liver (30, 31). Moreover, the different expression of tissue antigens between donors and recipients together with the clinical setting of induced immune-deficiency in recipients represent additional factors that remarkably worsen the impact of GvHD (32). In order to limit T cell alloreactivity, several haplo-HSCT platforms have been developed over the recent years (summarized in Table 1), including T-cell depleted (TCD) and T-cell replete (TCRep) approaches (5, 22, 25). Although the infusion of TCD grafts coupled with a mega-dose of CD34^pos^ peripheral blood HSCs (on average 10 × 10^6^ cells/kg body weight) ensures high engraftment rates associated with potent GvL effect and reduced GvHD, the small number of residual T lymphocytes administered in recipients are still able to induce high degrees of TRM and to delay IR with a subsequent increased rates of opportunistic infection onsets (5, 33). Hence, alternative and more efficient TCRep approaches able to better target alloreactive T cells have been developed in haplo-HSCT setting. These new protocols employ the infusion of high doses of post-transplant cyclophosphamide (PT-Cy), an immune-suppressant drug that is able to deplete *in vivo* all alloreactive and proliferating T cells (34). This new PT-Cy TCRep strategy showed since from the beginning very good clinical outcomes in term of engraftment, decreased GvHD and a faster kinetic of IR. Indeed, while donor T cell infused at the time of the transplant mediates a strong GvL in the first days soon after the administration of HSCs, the removal of those alloreactive and proliferating donor-derived T cells clones by PT-Cy limited the onset of GvHD afterward. These TCRep protocols have been then further optimized by infusing colony-stimulation factor (G-CSF)-primed grafts, by depleting *in vivo* selective T cell populations and by using a combination of other immune-suppressive agents (24, 35, 36).

Both the induced clinical condition of immune-deficiency early after allo- and haplo- HSCT and the delayed/aberrant IR facilitate the occurrence of opportunistic infections that greatly affect the quality and duration of life. Human cytomegalovirus (HCMV) is one of the most aggressive opportunistic microbes in allogeneic transplant including haplo-HSCT. Indeed, while HCMV infection is often asymptomatic or associated with mild flu-like symptoms in immune-competent hosts, its reactivation or *de novo* infection occurs in more than 50% of patients undergone haplo-HSCT within the first 3 months after the procedure and it remains a major cause of morbidity and mortality especially in TCD procedures (22, 37–45). Although the efficacy of the novel antiviral therapies decreased the incidence of HCMV infections/reactivations (46), this still represents one of main complications of allo-HSCT (47). In this regard, a careful selection of donors is recommended particularly within the haplo-HSCT setting, since their mismatch with the HCMV-serostatus of recipients greatly impacts the incidence and the virulence of HCMV reactivation (47). In particular, HCMV-seropositive recipients receiving a graft from HCMV-seronegative donors have the highest risks to develop HCMV reactivations. On the other hand, administering grafts from HCMV-seropositive donors increases the degree of OS in HCMV-seropositive patients receiving myeloablative conditioning (40). Hence, also the type of conditioning regimens plays a role in HCMV reactivations after allo-HSCT. The protective effect of HCMV-seropositive donors toward HCMV-seropositive recipient is also associated with the transfer of anti-HCMV specific T cell immunity (48). The frequency of primary infections in HCMV-seronegative recipients receiving a transplant from a HCMV-seronegative donor is very low since the reactivating viral strains generally origin from recipients, while their control is mediated by donor-derived alloreactive immune cells (45, 49, 50). However, a few other studies denied any significant impact of donor serostatus on HCMV reactivation in recipients undergone allo-HSCT (51, 52), thus leaving this important matter open for further discussion and clinical investigations. HCMV infections/reactivations also greatly affects the pattern of IR of both adaptive (53, 54) and innate immune cells (55, 56). Hence, it is conceivable that the kinetic of ILCs, NK and γδ T cell IR after haplo-HSCT as well as their effector-functions are somewhat influenced by HCMV infections/reactivations (55–58).

## Innate Lymphoid Cells

ILCs are a heterogeneous population of non-B and non-T lymphocytes that originate from common lymphoid progenitors. Since they lack adaptive antigen receptors, ILCs are able to rapidly produce and secrete pro-inflammatory and regulatory cytokines in response to local injuries, inflammation, infections or commensal microbiota perturbations (59–61). Similar to T cells, ILCs have been grouped into cytotoxic and helper lymphocytes and classified into three distinct sub-populations on the basis of their cytokines production and of the transcription factors involved in their development. These cell subsets are named ILC1, ILC2, and ILC3 and functionally mirror the CD4^pos^ T helper (Th)1, Th2, and Th17 cells, respectively. More recently, also NK cells have been grouped within ILC family and resemble the functions of CD8^pos^ cytotoxic T cells (59, 62–65).

ILC1 are mainly involved in interferon-γ (IFN-γ) production and represent potent effectors against bacterial and viral infections (61, 66–68). Despite sharing these functions with NK cells, ILC1 are currently considered a distinct subpopulation in terms of phenotype, function and development. Indeed, ILC1 are generally poorly cytotoxic and, unlike NK cells, are found at high frequency in tonsil and gut epithelium (i.e., intraepithelial ILC1) (69). Instead, ILC1 are rare in peripheral blood (PB) where they can be easily distinguished from NK cells due to their lack of CD56 and CD94 surface expression (63, 70, 71). ILC2 are also mostly tissue-resident lymphocytes and their effector-functions are triggered by interleukin (IL)-25 and IL-33 produced by epithelial cells or other immune cells in response to parasite infections or to allergen exposure. Following activation, ILC2 produce and secrete type 2 cytokines including IL-4, IL-5, IL-9, and IL-13 (62, 72–75). Moreover, ILC2 contribute to the resolution of inflammation by producing amphiregulin (AREG), a member of the epidermal growth factor that helps repairing damaged tissues (76). ILC3 are mainly resident in the gut lamina propria but have been also found in skin, lung and liver (77). Two different ILC3 subsets have been identified based on the expression of the Natural Cytotoxic Receptor (NCR) NKp44 in humans and NKp46 in mice. Both NCR^pos^/ and NCR^neg^/ILC3 subsets are able to produce IL-17, a cytokine crucial for fungal infection resistance. NCR^pos^/ILC3 can also secrete IL-22, an important cytokine that regulates the homeostasis of gut epithelium, prevents the dissemination of commensal bacteria and limits inflammatory response (78). Another subset of lymphocytes grouped within ILC family is represented by the so-called lymphoid tissue-inducer (LTi) cells that are mainly involved in lymphoid organogenesis in fetal life. However, LTi-like cells are present also in adult life where they facilitate the generation of secondary lymphoid organs (79). LTi/LTi-like cells also produce IL-22 and initiate protective immune responses against extracellular bacteria. However, these latter lymphocytes have been grouped separately from ILC3 since they have a unique transcriptional profile and are generated from distinct progenitors (80). Moreover, LTi/LTi-like cells are endowed with specialized functions related to adaptive immunity as they are involved in T and B cell development (79).

Despite their differences in term of phenotype and functions, several lines of evidence indicates that the helper-ILCs (i.e., ILC1, ILC2, and ILC3) have high degrees of cell plasticity, as each one of these three subsets can give rise to other members of the same family if cultured with the proper cytokine stimulation (81). Moreover, recent findings indicate that, although ILC1, ILC2, and ILC3 are mainly tissue-resident, they might traffic through the different organs by recirculating in the bloodstream. Indeed, gut-resident ILC2 can migrate into the lung and other peripheral tissues in response to helminthes or upon IL-25 stimulation to either fight the parasite infections or to contribute to tissue repair (82). This experimental evidence suggests that helper-ILCs, other than exerting anti-microbial responses and tissue remodeling in those organs where they reside under homeostatic conditions, can also mediate a protective role against tissue damage in different anatomic compartments following exposure to inflammatory stimuli. In the context of allo-HSCT, this phenomenon is highly relevant in the mucositis induced by chemo/radiotherapies, in the development of GvHD and in response to infections. However, little is known about the role(s) played by ILCs in the pathogenesis of hematologic malignancies as well as in the clinical outcomes of transplantation. Indeed, very few studies have addressed the role of immune- reconstituting ILC in the context of allo-HSCT (83), while their functions in haplo-HSCT remain still completely unexplored. Thus, in the next paragraphs we will summarize the evidence on ILCs in allo-HSCT setting.

### Immune-Reconstitution of Innate Lymphoid Cells

It has been recently disclosed that ILCs have a great clinical impact in patients affected by Acute Myeloid Leukemia (AML) either at disease onset or after chemo/radiotherapy and allo-HSCT (84, 85). In particular, there is a great reduction of circulating ILCs in AML, a phenomenon associated with a relative increase of ILC1 and a decrease of NCR^pos^/ILC3. The overall frequencies of PB NCR^pos^/ILC3 but not the ones of ILC1 are restored to normal levels in AML responders to chemotherapy. These quantitative changes of circulating ILCs in AML patients mirror their impaired abilities in producing IFN-γ and type 2 cytokines (85). Taken together, these data suggest that either leukemia burden or disease relapse markedly affect ILC development, a phenomenon also confirmed *in vitro* by co-culturing ILC precursors with AML blasts (86).

It has been also reported that conditioning regimens prior allo-HSCT deplete circulating ILCs that then undergo in recipients through a slow process of IR taking at last 6 months for a complete recover. In this setting, reconstituting ILCs show an increased expression of markers associated with tissue homing, such as the skin-homing receptors CLA and CCR10, the gut-homing molecules α4β7 and CCR6, the activation/tissue-residence marker CD69 and the cell proliferation nuclear protein Ki-67 (84). After 3 months from the transplant, the levels of circulating ILC2 are still strongly decreased compared to those of healthy subjects, while NCR^pos^/ILC3 outnumber the other ILC subsets (84). These data suggest that ILC3 play a major role in ILC IR after allo-HSCT. In line with this working hypothesis, a study showed that the high amounts of IL-22 produced by ILC3 can enhance both thymic regeneration and a more rapid T cell IR in a *IL2*^−/−^ mouse model receiving a TCD allo-HSCT (87).

It has been also reported that both conditioning regimens and different source of HSCs affects ILC IR after the transplant. This is of great importance in those children affected by severe combined immune deficiency (SCID) and carrying mutations of genes either encoding the common γ-chain subunit of IL-2 receptor or the tyrosine kinase JAK3. These patients lack all ILC subsets and experience an effective T cell IR following allo-HSCT only in the presence of myeloablative conditioning regimens (88). Instead, the administrations of cyclosporine or corticosteroids do not affect ILC IR (84). Another study showed in an *in vitro* setting that ILC3 IR is hampered by both pre- and post-transplant treatments with the mobilizing agent G-CSF (89). Moreover, it has been also reported that the generation of ILCs (especially NCR^pos^/ILC3) is much higher when culturing *in vitro* HSCs from bone marrow (BM) and umbilical cord blood rather than their counterparts from PB following mobilization with G-CSF (89).

#### Innate Lymphoid Cells and Graft vs. Host Disease

Several lines of evidence demonstrated that ILCs play a key role in limiting the onset of GvHD after allo-HSCT. In particular, it has been shown in murine models that ILC3 have a great impact in protecting recipient gut epithelial cells from alloreactive responses exerted by donor immune cells. This phenomenon is mediated by the ILC3 high production of IL-22 (90). Indeed, IL-22 deficient mice undergone allo-HSCT suffer from severe intestinal GvHD and intestinal barrier disruption, while the administration of IL-22 in transplanted wild type animals limits the onset of intestinal GvHD and enhances both intestinal stem cell recovery and epithelial cell regeneration (91). In humans, increased frequencies of circulating NCR^pos^/ILC3 early after allo-HSCT correlate with a lower incidence of intestinal GvHD. Notably, the ability to secrete high amounts of IL-22 by NCR^pos^/ILC3 exerts a key role in the regeneration of the mucosal gut barrier after immune depletion following allo-HSCT, thus protecting from GvHD onset (92, 93). Moreover, higher expressions on recipients' circulating ILCs of both CD69 and α4β7 markers before the transplant reduce the risk of developing GvHD and can serve as good prognostic factors (84). Even increased frequencies of CD69^pos^/ILC1 are associated with lower incidence of severe cutaneous GvHD since these cells express high levels of the skin homing markers CLA and CCR10. It has been also reported in murine models that type 2 cytokines play a protective role in GvHD development (92). Another reported mechanism protecting from GvHD is the ability of ILC2 to produce AREG that, in turn, boosts epithelial cell regeneration after the tissue damage induced by the conditioning regimens (76).

#### Innate Lymphoid Cells and Opportunistic Viral Infections

Although the role of ILCs in controlling infections in immune-competent individuals seem marginal, studies in immune-deficient mice showed that these innate lymphocytes can fight different pathogens (83, 94). However, very little if nothing is known in regard to their functional role in allo- and in haplo-HSCT setting. Since both T and B cell IR start to be effective and functional relevant only after a few months after haplo-HSCT, innate immune system certainly plays a key role in controlling opportunistic infections early after the transplant (19, 48, 95, 96). In this regard, while NK cells represent an immediate available source of IFN-γ in the bloodstream, ILC1 can provide large amounts of the same pro-inflammatory cytokine in tissues as reported in murine models of CMV, influenza, and Sendai infections (97, 98). Unlike ILC1, ILC2 are mainly involved in tissue damage repair during the resolution of the inflammatory process rather than in controlling the opportunistic infections (76, 99). Indeed, the proliferation and effector-functions of ILC2 are inhibited by both type I and II IFN that are largely produced during the course of viral infections (75, 100). Thus, high levels of IFN-γ produced by tissue-residence ILC1 not only control viral replication but also limit the dysregulation of ILC2 homeostasis.

## Natural Killer Cells

NK cells are innate lymphocytes playing a major role in the immune-surveillance mainly against cancer and viral infections without a prior antigen sensitization and through the signal delivered by large families of inhibitory and activating NK cell receptors (aNKRs and iNKRs) (101).

iNKRs recognize, as their natural ligands, “self” HLA-I molecules expressed on the surface of all nucleated cells, ensuring both the recognition of autologous targets and a certain threshold of immunologic tolerance especially at tissue levels. On contrary, tumor-transformed, viral infected, and heterologous cells lack or have reduced or express heterologous HLA-I molecules, respectively. NK cells can recognize these abnormalities on “non-self” and threatening targets due to the impaired or missing binding with iNKRs, whose downstream signaling is normally dominant over the activating stimuli driven by aNKRs in NK cells (“missing-self hypothesis”). The absence of this dominant inhibition shifts the balance toward NK cell activation via the engagement of aNKRs that binds their putative ligands on heterologous cell targets. These mechanisms trigger NK cell release of cytotoxic granules (i.e., perforin and granzymes) and secretion of anti-viral/pro-inflammatory cytokines for the clearance of both tumor and viral-infected cells (102–105).

The repertoire of NKRs is highly variable among different individuals and in different anatomic compartment and it is influenced by genetic factors, environmental exposure to non-self targets and tissue microenvironments (106, 107). Moreover, the phenotypic profiles of NK cells also depends by the so called “education/licensing” process that dictates the avidities of the interactions between iNKRs and their putative HLA ligands (108). The main classes of NKRs specific for HLA-I molecules include Killer Ig-like Receptors (KIRs) that recognize different HLA-A, -B, and -C allotypes (109) as well as the C-type lectin receptors CD94/NKG2A and CD94/NKG2C that bind the non-classical HLA-E molecules (110, 111). KIRs (known as CD158 molecules) represent a highly polymorphic family of NKRs that serve as regulators of development, tolerance and activation of NK cells (112). Interestingly, KIR superfamily includes both activating and inhibitory forms sharing homology in the extracellular domain, while differing for their cytoplasmatic tails. Activating KIRs (aKIRs) are characterized by a short intracellular domains that interact with adaptor signaling molecules carrying an Immunoreceptor Tyrosine-Based Activating Motif (ITAM) such as DAP-12 (113). On contrary, long cytoplasmatic tails containing Tyrosine-Based Inhibitory Motif (ITIM) distinguish inhibitory receptors (iKIRs) (109, 113, 114).

Similarly, the inhibitory C-type lectin receptor CD94/NKG2A is characterized by long intracellular tail containing ITIM motifs, while the trans-membrane domain of CD94/NKG2C interacts with the ITAM-containing adaptor molecule DAP-12 driving NK cell activation (115, 116). Among the other aNKRS driving the activation of NK cells there are the NCRs NKp30, NKp44, NKp46 together with the co-receptor NKp80 and 2B4 (117, 118).

CD16 (FCγRIII) is an immunoglobulin (Ig) receptor that, upon binding with the Fc portion of IgG antibodies, induces series of potent activating signals through the adaptor molecules CD3ζ and FcεRγ containing the activation ITAM motif. This down-stream pathway mediates the so-called antibody-dependent cell mediated cytotoxicity (ADCC) (119). The sequential expressions of CD16 together with KIRs, NCRs and C-type lectin receptors characterize the developmental stages, the effector-functions and the education of NK cells (120). The main steps of NK cell ontogenesis take place in BM niche starting from CD34^pos^ HSCs but, differently from helper ILCs, these innate lymphocytes are mainly enriched in PB (121). Indeed, under homeostatic conditions, NK cells account up to 10% of total circulating lymphocytes and represent an heterogeneous population that can be subdivided into two main subsets according to the surface expression of CD56 and CD16 (122). CD56^bright^/CD16^neg−low^ (CD56^br^) NK cells represent 5–15% of total circulating NK cells and are considered regulatory lymphocytes, as they produce high amounts of chemokines/cytokines and are involved in the cross-talk with other immune cells such as dendritic cells (DCs) and monocytes/macrophages (123–125). On the other hand, CD56^dim^/CD16^pos^ (CD56^dim^) NK cells are the largest NK cell subset in PB (up to 95%) and mainly exert cytototoxic functions via the secretion of lytic granules (104, 126–128). CD56^br^ and CD56^dim^ are also considered two sequential stages of NK cell maturation with the latter subset being the terminally-differentiation one characterized by shortest telomere length (120, 121, 129, 130). CD56^br^ NK cells usually show high levels of CD94/NKG2A, while almost lack KIRs (131). On contrary, CD56^dim^ NK cells acquire KIR expression and loose CD94/NKG2A, thus being fully licensed end-stage effector cells (115, 132). Despite intense efforts in better disclosing human NK cell ontogenesis, the mechanisms tuning the appearance of NKRs and the different NK cell developmental stages remain to be elucidated (120).

### NK Cell Immune-Reconstitution

Given the ability of NK cells to promptly mount effective alloreactive responses against tumor cells and pathogens, their kinetic and quality of IR certainly play important roles in determining the clinical outcome of allo- and haplo-HSCT. Indeed, delayed recoveries of these donor-derived alloreactive innate lymphocytes result in poor clinical outcomes of transplants (133, 134). As a matter of fact, NK cells are the first lymphocytes to appear soon after allo-and haplo-HSCTs and are essential for a better engraftment, to avoid tumor relapse and to limit the onsets of both GvHD and opportunistic viral infection. Moreover, the possibility to follow human NK cell IR in this unique *in vivo* setting is key in disclosing the several unknown mechanisms and patterns of their ontogenesis and differentiation (19, 135, 136). Regardless of the graft sources, NK cell chimerism in recipient is completely donor dependent after one month from haplo-HSCT. However, although the frequencies and absolute counts of circulating NK cells reach normal levels after few weeks post-transplant, their maturation and achievement of efficient effector-functions takes much longer (6, 15, 19, 130, 135, 137). Similar results have been observed also in recipients receiving HLA-matched HSCT, where reconstituting NK cells remain immature for more than 6 months after the infusion of HSCs. These phenomena are associated with functional defects that do not ensure an optimal protection against HCMV infections/reactivations, GvHD onset and tumor relapse in the first year after HSCT (138).

Reconstituting NK cells derive from CD34^pos^ progenitors rather than from already mature NK cells infused with the graft. Indeed, the PT-Cy eliminates proliferating alloreactive NK cells in haplo-HSCT as they have an even higher proliferation rate compared to T cells in the first days after the graft infusion and before the Cy administration. The 2^nd^ wave of proliferating donor-derived NK cells occurs after 15 days from haplo-HSCT and these new innate lymphocytes display an immature phenotype, thus confirming that they are *de novo* generated from donor HSCs (96). Indeed, CD56^br^ NK cell subset appears much earlier that terminally differentiated CD56^dim^, while the NK cell surface distribution of both CD56 and CD16 return similar to that of healthy donors only several months later (6, 19, 96, 120, 139–141). Unexpectedly though, we recently reported that the subset of reconstituting donor-derived NK cells expanded at the highest frequency in the first weeks after haplo-HSCT is characterized by an unconventional CD56^dim^/CD16^neg−low^ phenotype (unCD56^dim^). This neglected NK cell population is present at very low frequency under homeostatic conditions, but plays a key role in the IR and in the clinical outcome of haplo-HSCT. In particular, although armed to be cytotoxic and carrying large amounts of perforin and granzymes, unCD56^dim^ NK cells are highly defective in their killing activities due to the transient high expression of CD94/NKG2A receptor. Hence, this C-lectin type receptor functions as an inhibitory checkpoint that renders donor-derived unCD56^dim^ NK cells anergic against residual tumor cells, recipients T cells and Antigen Presenting Cells (APC). This NK cell status early after haplo-HSCT makes recipients more at risk to undergo tumor relapse and to develop acute (a) GvHD. Similar transient high surface levels of CD94/NKG2A have been observed also on terminally-differentiated and cytotoxic CD56^dim^ NK cells that start to reconstitute from the 2^nd^ month after the transplants and subsequent to the appearance of unCD56^dim^ NK cells (19, 135, 139, 142). This gained knowledge paved the ground for a novel therapeutic approach targeting CD94/NKG2A in order to unleash NK cell cytotoxicity in haplo-HSCT.

### NK Cells and Graft vs. Host Diseases

The HLA-mismatch between donor and recipient cells allow donor-derived and alloreactive NK cells to both limit the onset of GvHD and to prevent graft rejection in allo- and haplo-HSCT (143, 144). Indeed, several studies directly correlated an efficient NK cell IR in allogeneic transplant with the reduced incidence of relapse as well as with decreased rates of opportunistic infections in the presence of lower TRM and increased OS (134, 145, 146). In contrast, low frequencies of NK cells in the first weeks after allo-HSCT are associated with increased non-relapse mortality, shorter OS and higher degrees of opportunistic infections (133, 145). This clinical evidence underline the importance of NK cell IR in shaping the clinical outcomes of allogeneic transplants and its possible exploitation for developing novel therapeutic strategies (2, 135, 147). However, the exact NK cell-mediated mechanism preventing GvHD onset are not yet fully elucidated. One working hypothesis is that alloreactive NK cells could limit GvHD by killing donor T cells via the NKG2D-mediated recognition of stress-induced NKG2D-ligands on activated T lymphocytes (148, 149). Another study claimed that high frequency of NK cells in the first weeks after HSCT might prevent T cell proliferation through IL-10 production (150). Conversely, it has been also reported that NK-cell production of pro-inflammatory IFN-γ could promote tissue damage and consequent GvHD (151). Notably, also the quality of NK cells IR greatly affects the occurrence of GvHD after allo-HSCT. Indeed, higher surface levels of CD94/NKG2A on NK cells have been reported to limit aGvHD *in vivo* by inhibiting T cell proliferation and activation (152). Furthermore, increased frequencies of CD94/NKG2C^pos^ NK cells are associated with a lower incidence of GvHD in allo-HSCT (153).

Even the NK cell maturation stage is important, as a recent report showed that those haplo-HSCT recipients developing GvHD display a more differentiated and activated NK cell phenotype (154). This evidence has been also further corroborated by other studies reporting that a reduction of circulating CD56^br^ NK cells in the first 2 months after allo-HSCT is associated with higher incidence of aGvHD. This latter clinical correlation was so evident in the recruited cohorts of patients receiving allo-HSCT to be proposed as an early prognostic factor to predict GvHD (141, 143). Moreover, a higher ratio of T/NK during IR after phase correlates with a higher risk to develop both acute and chronic GvHD in haplo-HSCT (8).

Remarkably, the potential clinical benefits of reconstituting NK cells in haplo-HSCT might be influenced by pre- and post-conditioning treatments. In this regard, many studies performing adoptive transfer of NK cells after haplo-HSCT showed a reduced risk in aGvHD induction (151). Moreover, GvHD prophylaxis with Mycophenolate Mofetil has been demonstrated to inhibit NK cell proliferation and effector-functions (155, 156), thus affecting the NK cell mediated control of GvHD and opportunistic infections.

#### NK Cells and Viral Infections

The occurrence of an optimal quantitative and qualitative NK cell IR in haplo-HSCT is key for hampering the onset of life-threatening opportunistic infections. Indeed, lower frequencies of circulating donor-derived NK cells are associated with higher susceptibilities to develop viral infections, mainly HCMV (157). In turn, HCMV infections/reactivations are also able to influence NK cell homeostasis and differentiation by inducing the expansion of the CD56^neg^/CD16^pos^ (CD56^neg^) NK cell subset (158, 159). While poorly represented in healthy individuals, CD56^neg^ NK cells are present at high frequencies in active and chronic HIV-1 and HCV infections (58, 160, 161) and display impaired effector-functions due to their abnormal repertoire of NKRs (162, 163). Indeed, CD56^neg^ NK cells are defective in the clearance of viral infections and express markers of cell exhaustion of their surface (164, 165). However, the ontogenesis and the impact of CD56^neg^ NK cells in determining the clinical outcomes of allo- and haplo-HSCT are still being debated. Recent studies revealed that HCMV infections/reactivations are beneficial rather than detrimental on NK cell recovery upon haplo-HSCT. In particular, it has been reported that this virus can accelerate NK cell maturation and shape their NKR repertoire in haplo-HSCT by inducing the expansion of terminally-differentiated and alloreactive CD56^dim^ NK cells which, in turn, exert potent GvL effects (166). Indeed, upon HCMV infections/reactivations, CD56^dim^ NK cells acquire a mature NKG2C^pos^/CD57^pos^/NKG2A^neg^/KIR^pos^ phenotype, thus becoming fully licensed to efficiently exert anti-viral and anti-tumor properties (i.e., production of IFN-γ and Tumor Necrosis Factor (TNF)-α) (167–169). On the contrary, NK cells from haplo-HSCT patients that do not experience HCMV infections/reactivations retain an immature phenotype characterized by high expressions of CD94/NKG2A (170).

These HCMV-induced NKG2C^pos^/CD57^pos^/NKG2A^neg^/KIR^pos^/CD56^dim^ NK cell subset can persist even after 1 year from haplo-HSCT and show higher effector-functions when re-encountering the same antigen or following a proper activation with specific pro-inflammatory cytokines. These data suggest that HCMV infections/reactivations drive the expansion of NK cells with adaptive properties (167, 170–172). Similar features have been reported in murine models *in vivo*, where the murine CMV (MCMV) infection is responsible for the expansion of the so-called “memory-like” NK (ml-NK) cells that specifically recognize the viral glycoprotein m157 through the activating receptor Ly49H (173, 174). However, neither a univocal phenotype nor the receptor(s) able to specifically bind HCMV antigens have been clearly defined in human ml-NK cell and this is a matter currently being highly investigated in several models *in vitro* and *ex vivo*. In this regard, NKG2C has been proposed as the best putative candidate binding HCMV antigens, since those NK cells expressing this aNKR are the ones preferentially expanded following this viral infection (175, 176). In this regard, it has also been reported that the HCMV-encoded UL40 protein stabilizes HLA-E surface expression on target cells, thus favoring the recognition of viral-infected via the NKG2C/HLA-E interactions (159, 177). Moreover, another study claimed that proliferation/expansion of NKG2C^pos^ NK cells requires additional signaling pathways including the one mediated by IL-12 produced by autologous monocyte (178). Despite all the above-mentioned experimental evidence, the primary role of NKG2C in the homeostasis and functional relevance of ml-NK cells is still unclear. Indeed, other subsets of NKG2C^neg^/KIR^pos^ NK cells are also expanded in response to HCMV infection and they are able as well to recognize viral-infected cells (106), thus suggesting the existence of additional aNKRs (i.e., KIRs) involved in the expansion of human ml-NK cells (179). Furthermore, NKG2C-deficient individuals can mount equivalent adaptive NK cell response against HCMV (180). In agreement, in patients receiving cord blood grafts from NKG2C^−/−^ donors, HCMV infection is still able to promote NK cell maturation in the absence of this activating C-lectin type molecule. This latter experimental evidence further supports the current working hypothesis that other NKRs such aKIRs play a central role in the generation of ml-NK cells (181).

More recently, other studies demonstrated that the generation of ml-NK cells is associated with epigenetic reprogramming through a specific reconfiguration of adaptor molecules including tyrosine kinase SYK, the intracellular adaptor EAT-2, and the transmembrane adaptor protein FcεRγ. The gene expression of these three factors is regulated by the transcription factor promyelocytic leukemia zinc finger (PLZF), which is downregulated in the majority of ml-NK cells upon HCMV infections. As a matter of fact, the reduced expression of at least one of the above-mentioned signaling proteins is observed in the 50% of the HCMV-seropositive donors. Moreover, the reduced levels of PLZF also decreases the expression of IL-12 and IL-18 receptors, thus lowering NK cell responsiveness to these pro-inflammatory cytokines. The lack of FcεRγ, SYK, and EAT-2 in mature CD56^dim^ NK cells is also correlated with the expansion of NKG2C^pos^ NK cells upon HCMV infection (182–185). In this regard, CD56^neg^ NK cells expanded in those patients receiving umbilical cord blood transplant and experiencing HCMV infection/reactivation are characterized by the downregulation of FcεRγ (186). In addition to the downregulation of PLZF, FcεRγ, SYK, and EAT-2, ml-NK cells share with cytotoxic CD8^pos^ T cells similar genome-wide DNA methylation patterns (182), thus suggesting the existence of epigenetic determination programs associated with HCMV infections. Notably and similar to memory Th1 lymphocytes, the increased production of IFN-γ by ml-NK cells correlates with a stable demethylation of conserved non-coding sequence 1 of the *IFNG* locus (187).

Although there is a phenotypic heterogeneity of ml-NK cells following HCMV exposure, their rapid maturation in response to the viral challenges could favor not only the control of infection, but also NK cell alloreactivity against residual tumor cells (188). Hence, HCMV infection can represent a “natural” tool to generate ml-NK cells to then use for adoptive cellular immunotherapies (132, 189, 190). In this regard, newborn mice challenged with MCMV showed that ml-NK cells undergo expansion, release cytokines and provide a protective anti-tumor immune response in adoptive cell transfers (173). In humans, the expansion and the functional relevance of NKG2C^pos^ ml-NK cells in HSCT recipients experiencing *de novo* viral infection or undergone HCMV reactivations also depends from donor serostatus. Indeed, the *in vivo* expanded NKG2C^pos^ ml-NK showed higher cytokine productions in those recipients receiving grafts from HCMV-seropositive donors compared to their counterparts originated from grafts of HCMV-seronegative donors. However, NKG2C^pos^ ml-NK cells also expand in the absence of detectable HCMV viremia when both donor and recipient are HCMV-seropositive. These data suggest that also human NKG2C^pos^ ml-NK cells are transplantable and require exposure to either active or latent (subclinical) HCMV antigens in the recipients for the expansion of alloreactive NK cells from seropositive donors (191). Moreover, NKG2C^pos^ ml-NK cells are able to produce high levels of IFN-γ following *in vitro* co-culture with K562 erytroleukemia cell line, thus supporting their high potential in GvL effect (192). Consistent with these findings, the adoptive transfer of donor-derived or cytokine-induced (i.e., activation with IL-12, IL-15, IL-18) ml-NK cells induces in the recipients affected by refractory AML the expansion of NK cells producing high levels of IFN-γ when encountering tumor cell targets (172).

Taken together these results suggest that ml-NK cells can be potentially exploited in order to both better control HCMV infection/reactivation and to enhance GvL (193).

## γδ T Cells

γδ T cells are a group of unconventional T cells that bridge the gap between innate and adaptive immunity. Similar to αβ T cells, γδ T cells develop in the thymus and express a somatically rearranged T cell receptor (TCR) consisting of a TCR-γ and a TCR-δ chains (65, 194–196). In humans, γδ T cells normally account for the 1–10% of circulating T lymphocytes, while in mucosal tissues and skin they constitute the major subset of resident T cells (194, 196). Different γδ T cell subsets can be identified based on the Vδ expression (Vδ1, Vδ2, Vδ3, and Vδ5) (195, 197). Under homeostatic conditions, 95% of circulating γδ T cells express Vδ2 TCR paired with Vγ9 chain, whereas in mucosa and skin γδ T cells mostly express Vδ1 or Vδ3 TCRs paired with various Vγ chains (195, 198–200).

γδ T cells are rapid responders to pathogens and tumor-transformed cells, since they do not require further peripheral maturation or extensive clonal expansion to initiate their effector-functions (194). Therefore, γδ T cells allow a prompt immune-surveillance in a MHC-independent manner through the recognition of a diverse array of antigens including peptides, sulfatides and phospholipids (194, 196, 199, 201, 202). Moreover, the γδ TCR can bind CD1d expressed by APC loaded with glycolipids and microbial lipids (203). In addition to their TCR, γδ T cells express an array of pattern-recognition receptors, such as toll-like receptors (TLRs) (201, 204), activating and inhibitory NKRs (201, 205, 206), the NCRs NKp30 and NKp44 (206, 207), the aNKR DNAM-1, the Fc receptor CD16 as well as the C-type lectin-like receptors NKG2D and CD94/NKG2A (195, 206, 208, 209). The presence of such receptor repertoire suggests a tight regulation of the TCR-mediated activity through an interplay between activating and inhibitory signaling downstream pathways (206).

Upon their activation, γδ T cells secrete high levels of Th1 cytokines (i.e., IFN-γ and TNF-α) modulating the responses of other neighboring immune effectors which, in turn, induce monocyte-derived DC maturation/activation and enhance antigen-specific αβ T cell responses (194, 195). Moreover, γδ T cells are able to directly lyse target cells by the release of granzymes and perforin and the engagement of FAS and TRAIL death receptors (195, 197, 210). As consequence of their high heterogeneity, γδ T cells are implied in diverse biological functions. First, these cells exert anti-tumor activities against various types of solid tumors and hematological malignancies (211). Since they represent the most abundant population among epithelial-resident lymphocytes in mucosal tissues and skin, γδ T cells are also the first line of defense against pathogens in these anatomic compartments (211, 212). Finally, several γδ T cell subtypes are involved in the induction of transplant immune-tolerance both in solid organ transplantation and in allo-HSCT (211, 213).

### γδ T Cell Immune-Reconstitution

The growing interests on the role of γδ T cells IR in HSCT arose from their potential ability to perform GvL effects and fight opportunistic infections in the absence of GvHD (205, 211, 214). Indeed, pediatric and adult patients undergone haplo-HSCT and showing a long-term disease-free survival (DFS) were coupled with high frequencies of circulating γδ T cells (215, 216). γδ T cells are also the predominant T cell population reconstituting early after haplo-HSCT, with the Vδ2 cells showing a faster recovery compared with B and T lymphocytes in the PB of recipients receiving αβ and CD19 depleted grafts. In particular, it has been shown that the recovery of the complimentary determinant region 3 (CDR3) of the TCR δ chain is almost completed after 2 months from haplo-HSCT (25, 28, 29, 48, 95, 217). In the context of allo-HSCT, the majority of both donor-derived Vδ1 and Vδ2 cell subset recovering in the first weeks have a CD27^pos^/CD45RA^neg^ Central Memory (CM) phenotype and contribute to ensure an early protection against viruses, bacteria and residual tumor cells that survived the conditioning regimes (65). The current working hypothesis of a peripheral expansion of graft-derived mature γδ T cells is further supported by experimental evidence indicating that the same γδ T cell clones found in the donor are present in the recipient after the transplant (218). Later on, within a range of 14–60 days post-transplantation, the frequency of CM γδ T cells progressively decreases and it is counterbalanced by increase frequencies of naïve CD27^pos^/CD45RA^pos^ γδ T cells originated from donor infused HSCs (28, 65). This latter *de novo* generation of reconstituting γδ T cells is confirmed by the fact that, while the repertoire of the γ and δ chains is qualitatively comparable between donors and recipients, their clonotype is different (57).

γδ T cell IR after allo- and haplo-HSCT can be influenced by different variables including the conditioning regimen, the administration of immuno-suppressive agents, the GvHD prophylaxis and the onset of opportunistic infections (211). In this regard, it has been reported that stem cell mobilization with G-CSF in allo-HSCT induces higher frequencies of Vδ1 T cells endowed with potent alloreactivity against AML blasts (214). Moreover, also donor/recipient characteristics (i.e., gender, age, disease type, and graft source) affect γδ T cell IR too. Indeed, patients receiving a transplant from either matched related (MRD) or haplo-related donors have significant differences in the recovery of γδ T cells compared to matched unrelated donor (MUD) (215).

#### γδ T Cells and Graft vs. Host Diseases

It has been reported in allogenic HSCT that patients developing aGvHD show an increased frequency of reconstituting γδ T cells (219). However, this evidence has been denied by more recent findings indicating that absolute counts of γδ T cells do not influence the incidence and the severity of GvHD (65, 215). Instead, higher frequencies of donor-derived γδ T cells in the grafts seem to protect against the development of severe aGvHD (220). Similarly, patients receiving a TCD haplo-HSCT and showing increased frequencies of γδ T cells undergo longer DFS and OS compared to those with normal/decreased immune-reconstituting γδ T cells. These data corroborate the current consensus stating that γδ T cells can facilitate GvL effect without inducing GvHD (196, 216, 221, 222).

#### γδ T Cells in Viral Infections

The occurrence of high frequencies of reconstituting γδ T cells early after haplo-HSCT also protect from bacterial infections and show a decreased incidence of both viral and fungal infections (215). Indeed, pediatric patients, receiving αβ TCD grafts in haplo-HSCT setting have both reduced numbers of γδ T cells at day 30 post-transplant and higher incidence of HCMV infections/reactivations (65). At the same time, opportunistic infections can also shape the homeostasis and maturation of these cells (28, 55, 57, 195, 223). Indeed, patients undergoing allo-HSCT and experiencing HCMV reactivations display a preferential proliferation of specific Vδ1 and Vδ3 T cell clones, thus suggesting that γδ T cells are capable of adaptive responses through an oligoclonal selection of specific TCR repertoires (57). In particular, HCMV reactivation in haplo-HSCT patients has been associated with a specific expansion of terminally differentiated cytotoxic Vδ1 T expressing the effector memory CD45RA^pos^/CD27^neg^ (TEMRA) phenotype (28). This HCMV-induced expansion of TEMRA γδ T cells also enhance their anti-tumor functions both against hematological (28, 223) and solid (224) tumor cell targets *in vitro*. Taken together, these results suggest that the adoptive transfer of HCMV-specific Vδ1-donor γδ T cells can be used as a possible alternative to the common infusion of HCMV-specific αβ T cells (225). Indeed, this novel approach could promote viral immunity, protect from HCMV-related complications while contribute to prevent from leukemic relapses (214).

## Novel Therapeutic Strategies to Improve IR Upon HSCT

**Figure 1:**
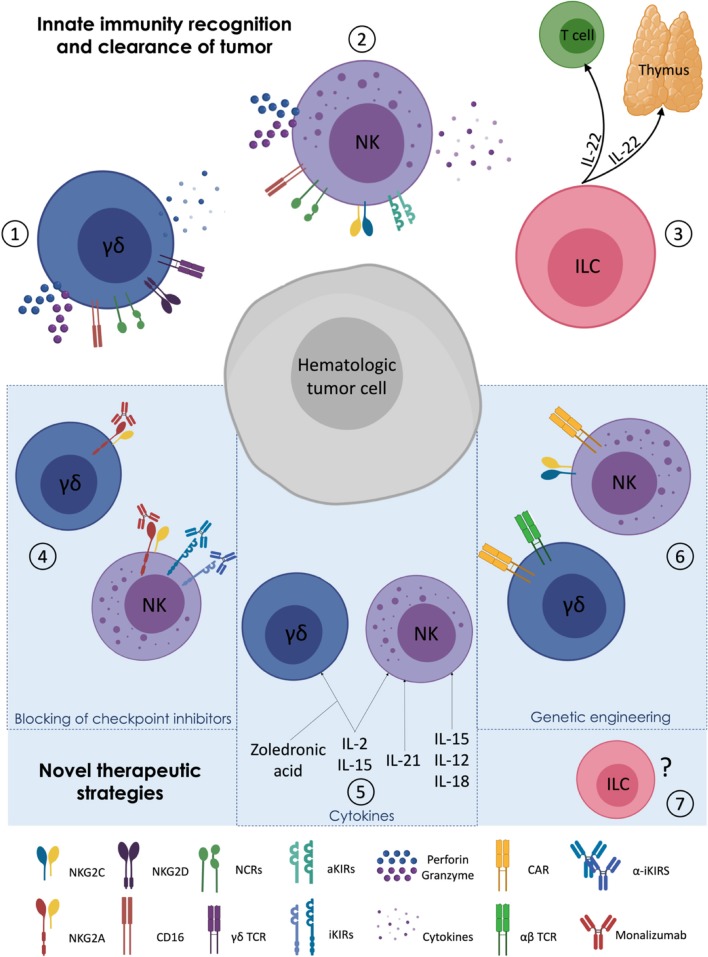
Targeting γδ T, Natural Killer, and Innate Lymphoid cells in haplo-HSCT. (1–3) MHC-independent activation of innate immune cells: γδ T lymphocytes (1) and NK cells (2) can kill hematologic tumors by direct cytotoxicity and cytokine secretion. Innate Lymphoid cells (ILCs and ILC3 in particular) (3) play an indirect role in the clearance of tumors cells by improving both thymic regeneration and T cell maturation via their secretion of IL-22. (4–6) Novel therapeutic strategies implemented to enhance NK and γδ T cell alloreactivity against cancer: administration of monoclonal antibodies (mAbs) against NK cell inhibitory checkpoints (4); use of cytokines and zoledronic acid to activate γδ T cells (5); CAR editing and genetic engineering of γδ T and CAR on NK cells (6). (7) *Ad hoc* manipulation/editing/engineering of ILCs in transplant setting have not yet been explored.

The early protection and the limited side effects following HSCT render innate immune system a particularly attractive tool for adoptive cell therapy strategies. In this context, several approaches have been recently developed to improve NK and γδ T cell IR and to enhance their reactivity against cancer. These new therapeutic strategies include the targeting of checkpoint inhibitors, the stimulation with activating cytokines and genetic engineering of immune cells (Table 2) (Figure 1).

**Table 2:** Clinical trials targeting NK/γδ T cells in HSCT to cure patients with hematologic malignancies.

**Therapeutic approach**	**Study title**	**Study** **phase/** **status**	**Hematologic disease investigated**	**Cell sources/targets**	**Drug/CAR construct**	**NCT number**
Blocking mAbs	Study of a humanized antibody initiated 2 months After an HLA matched allogenic stem cell transplantation	Phase I/ recruiting	Hematologic malignancies (AML, ALL, MDS, MM, CLL, CML, myeloproliferative neoplasm, HD, NHD)	T, NK	Anti-NKG2A mAb, IPH2201, monalizumab	02921685
Blocking mAbs	Combination study of IPH2201 with Ibrutinib in patients with relapsed, refractory, or previously untreated chronic lymphocytic leukemia	Phase I-II/ active, not recruiting	Relapsed and refractory CLL	T, NK	Anti-NKG2A mAb, IPH2201, monalizumab	02557516
Blocking mAbs	Study on the anti-tumor activity, safety, and pharmacology of IPH2101 in patients with smoldering multiple myeloma	Phase II/ completed with results (226, 227)	Smoldering MM	NK	Anti-KIR mAb, IPH2101, Lirilumab	01222286
Blocking mAbs	Evaluation of activity, safety and pharmacology of IPH2101 a human monoclonal antibody in patients with multiple myeloma	Phase II/ completed with results (226, 227)	MM	NK	Anti-KIR mAb, IPH2101, Lirilumab	00999830
Blocking mAbs	A safety and tolerability extension trial assessing repeated dosing of anti-KIR (1-7F9) human monoclonal antibody in patients with acute myeloid leukemia	Phase I/ completed	AML	NK	Anti-KIR mAb, IPH2101, Lirilumab	01256073
Cytokines and drug stimulation	Interleukin-21 (IL-21)- expanded natural killer cells for induction of acute myeloid leukemia	Phase I-II/ recruiting	AML	NK	IL-21	02809092
Cytokines and drug stimulation	Donor natural killer cells in treating patients with relapsed or refractory acute myeloid leukemia	Phase I-II/ recruiting	AML	NK	IL-21	01787474
Cytokines and drug stimulation	Natural killer cells before and after donor stem cell transplant in treating patients with acute myeloid leukemia, myelodysplastic syndrome, or chronic myelogenous leukemia	Phase I-II/ recruiting	AML, MDS, CML	NK	IL-21	01904136
Cytokines and drug stimulation	Cytokine induced memory-like NK cell adoptive therapy after haploidentical donor hematopoietic cell transplantation	Phase II/ recruiting	AML	NK	IL12-IL15-IL18	02782546
Cytokines and drug stimulation	Cytokine-induced memory-like NK cells in Patients With Acute Myeloid Leukemia (AML) or Myelodysplastic Syndrome (MDS)	Phase I–II/ recruiting	AML	NK	IL12-IL15-IL18	01898793
Cytokines and drug stimulation	Zoledronic acid in combination with interleukin-2 to expand Vγ9Vδ2 T cells after T-replete haplo-identical allotransplant	Phase I/ recruiting	Hematologic malignancies	γδ T	Zol+IL2	03862833
Cytokines and drug stimulation	Expanded/activated gamma delta T-cell infusion following hematopoietic stem cell transplantation and post-transplant cyclophosphamide	Phase I/ not recruiting	AML, CML, ALL, MDS	γδ T	CliniMACS-Prodigy technology	03533816
Genetic engineering	Genetically modified haploidentical natural killer cell infusions for B-lineage acute lymphoblastic leukemia	Phase I/ completed	ALL	CAR-NK	Anti-CD19-BB-zeta	00995137
Genetic engineering	Pilot study of redirected haploidentical natural killer cell infusions for B-lineage acute lymphoblastic leukemia	Phase I/ suspended	ALL	CAR-NK	Anti-CD19-BB-zeta	01974479
Genetic engineering	Umbilical and Cord Blood (CB) derived CAR-engineered NK cells for b lymphoid malignancies	Phase I–II/ recruiting	ALL, CLL, NHL	CAR-NK	Anti-CD19-CD28-zeta-2A-iCasp9-IL15-transduced CB NK cells	03056339

*ALL, Acute lymphoid Leukemia; AML, Acute myeloid Leukemia; CB, Cord blood; CLL, Chronic lymphoid Leukemia; CML, Chronic myeloid Leukemia; HL, Hodgkin lymphoma; IL, interleukin; MDS, Myelodisplastic syndrome; NHL, Non-Hodgkin lymphoma; mAb, monoclonal antibody; MM, multiple myeloma; Zol, Zoledronic acid*.

## Checkpoint Inhibitor

NK cells and γδ T lymphocytes share several receptors including NCRs and iNKRs as CD94/NKG2A (195, 206, 209). The use of monoclonal antibodies (mAbs) against inhibitory immune checkpoints represents a promising therapeutic approach for both hematologic and solid tumors (228, 229). Of particular relevance, the blockade of NKG2A binding to HLA-E has been demonstrated to unleash the effector-functions of both T and NK cells in different kind of tumors (230–233). These encouraging results have driven the development of humanized IgG4 anti-NKG2A mAb (IPH2201, monalizumab), currently under investigation in many clinical trials for the treatment of solid tumors (clinicaltrials.gov) (Table 2). Conversely, only one phase I clinical study is now investigating the potential role of IPH2201 in hematologic malignancies after HLA-identical transplantation (NCT02921685). In this regard, our recent data demonstrate that there is a clear clinic indication to extend the IPH2201 administration early after haplo-HSCT, thus targeting those hypo-functional NK cells expressing high levels of NKG2A with the aim of enhancing their alloreactivity (19). Moreover, given the fast recovery of γδ T lymphocytes following haplo-HSCT, the post-transplant infusion of IPH2201 could also positively impact their anti-tumor responses in synergy with NK cells before the acquisition of a full functional competence of the adaptive immune response (i.e., T and B cells).

Among other receptors regulating NK cell missing-self responses, KIRs cover an important place. Indeed, their clinical impact have been firstly shown in AML patients undergoing haplo-HSCT where the mismatch between KIRs and their ligands in the recipient has been exploited to promote alloreactive NK cell-mediated GvL effect (135). In this context, therapeutic anti-KIR mAb (IPH2101, 1-7F9, lirilumab) has been generated and its administration showed positive outcomes in AML and multiple myeloma (MM) patients (Table 2) (226, 227).

## Cytokines

As anticipated, NK cell anti-tumor responses are finely governed by an array of NKRs tuning their balance between inhibition and activation. This gained knowledge allowed to implement several protocols of *in vitro* NK cell manipulation that use cytokines to regulate the aNKR repertoire, thus boosting their killing ability against tumor targets (Table 2).

IL-2 and IL-15 represent the first molecules used to induce the proliferation and increase the cytotoxic potential of both T and NK cells for adoptive cell transfer therapies in different tumor settings (234). Later on, IL-21, another cytokine involved in NK cell maturation (235) also gained clinical relevance for the treatment of hematologic malignancies. Indeed, a recent phase I clinical trial using K562-based feeder cells expressing membrane-bound chimeras of IL-21 (mbIL21) was conducted in patients affected by AML/myelodysplastic syndrome and demonstrated that the infusion of *ex vivo*–expanded NK cells from BM haplo-donor could control tumor relapse without major toxicity (236). Other clinical trials exploiting the same technology are currently ongoing for AML in haplo-HSCT setting (Table 2) (NCT02809092, NCT01787474, NCT01904136). In order to optimize NK cell expansion and effector-functions, other experimental approaches also tested the combination of different cytokines. In particular, the stimulation with IL-15, IL-12, and IL-18 together drive the expansion of a particular subset of NK cells displaying adaptive traits similar to those of ml-NK cells re-challenged by HCMV (172). The adoptive transfer of these donor-derived and cytokine-induced ml-NK cells in patients affected by refractory AML is associated with higher levels of IFN-γ encountering and eliminating tumor cell targets (172). Two clinical trials are currently administering cytokine-induced ml-NK cells in AML patients undergone haplo-HSCT (Table 2) (NCT02782546, NCT01898793).

The combination of IL-2 and IL-15 either alone or in synergy with other stimulant agents have been extensively used also to expand γδ T cells (237). In this regard, one of the more promising protocols is represented by *in vivo* post-transplant administration of Zoledronic Acid (ZA) that improves the cytotoxicity of γδ T cells against leukemic cells (Table 2). This latter strategy relies on the use of ZA and IL-15 to expand terminally-differentiated and anti-tumor CD45RO^neg^/CD27^neg^ effector memory (TEMRA) Vδ2 cells. In this setting, the use of IL-15 is meant also to simultaneously boost the cytotoxicity and the proliferation of NK cells, thus targeting the two main anti-cancer effectors at the same time (28, 238, 239). In haplo-HSCT platforms, two very recent phase I studies propose to expand/activate γδ T cell prior (NCT03533816) or after (NCT03862833) cell infusion to provide innate GvL responses and to limit the onset of GvHD (Table 2).

## Genetic Engineering

Genetic manipulation of immune cells allows the generation of highly specific anti-tumor effectors effectively targeting several tumor antigens. The introduction of chimeric antigen receptor (CAR)-T cells in HSCT opened new insight for the treatment of hematologic malignancies. Despite the very good clinical outcomes given by autologous CAR-T cell therapies against several tumors (240–243), the occurrence of life-threatening side effects such as tumor relapses (240, 244) and higher frequencies of GvHD and cytokine release syndrome onsets (245) have arisen major limitations in the use of allogeneic CAR-T cells. In this regard, engineering CAR-NK and CAR-γδ T cells may provide alternative procedures to improve their anti-tumor potentials, while overcoming allogeneic CAR-T cell therapy obstacles (Table 2) (139, 246–249). Notably, CAR-NK cells and CAR-γδ T cells retain the expression of their NKR repertoire and γδ TCR, respectively (214, 250). Hence, they can recognize tumor targets by their native receptors independently from CAR-restriction, thus reducing antigen-driven escape of tumor cells and further increasing their killing activities. CAR-NK cells are also characterized by relatively short life-span. If this latter feature certainly limits NK cell cytotoxicity over the time after transplantation, it can then prevent long-term side effects (such as cytopenia) that are observed upon CAR-T cell infusion (251).

A multitude of preclinical studies have tested the efficacy of CAR-NK cells against a variety of target antigens such as CD19 (252, 253) and CD20 (254, 255) for hematological malignancies as well as solid tumors. Another methodology used to promote the persistence of CAR-NK cell is to incorporate genes for IL-2 (256, 257) or IL-15 (258) within the CAR construct to constantly provide cytokine support to the CAR-transduced cells. In particular, this approach showed improved tumor control and prolonged survival in a mouse model of Raji lymphoma (258). These encouraging pre-clinical data opened new insights for the transfer of such protocols into human clinical trials such as the one that is optimizing the dose of IL-15-transduced CAR-NK cells for the treatment of B cell lymphoma (Table 2) (NCT03056339). Finally, genetic engineered CAR-NK cells mimicking ml-NK cells have been obtained redirecting NKG2C-mediated NK cell responses against cells expressing HLA-E. This protocol allows to overcome the dominant NKG2A-mediated inhibition, while boosting CAR-redirected NK cell activation via NKG2C (259).

Besides NK cells, also γδ T cells have been engineered against tumor targets using CAR technology (260). However, although CAR-γδ T cells were firstly introduced in 2004 (249), relatively few studies report their benefic potential in the treatment of hematologic and solid tumors.

Among these trials, PB-derived Vg9Vd2 T cells transduced with retroviral vectors encoding either disialoganglioside GD2- or CD19-specific CARs showed a higher capacity to secrete antigen-specific IFN-γ and to exert potent cytotoxicity against GD2^pos^ neuroblastoma cells and CD19^pos^ leukemic blasts *in vitro* (249). Furthermore, γδ T cells can be also transduced with exogenous αβ TCR directed against tumor associated antigens (214, 261). However, no clinical trials using CAR-γδ T cells have been initiated yet.

## Concluding Remarks

Great efforts have been put in place to ameliorate the clinical outcome of allo-HSCT, to find an ideal donor for every patient in need and to limit the life-threatening complication of this transplant procedure. The development of haplo-HSCT platforms certainly represents a great step forward on these matters, although quite a few side effects, including the occurrence of GvHD and opportunistic infections, still affect the quality and the duration of life of these patients. In this regard, the quantity and quality of IR play a central role and require a deep understanding of all the mechanisms tuning the kinetic and the effector-functions of those immune cells that can better control the onset of tumor relapse, GvHD, and opportunistic infections. In this context, innate immune responses are key as they act immediately after the transplant. Several experimental and clinical studies clearly highlighted the importance to boost both adaptive and innate IR, ameliorate anti-tumor alloreactivity and develop alternative immunotherapy weapons against cancer.

The advances of current technologies have optimized the *ex vivo* expansion/activation of immune effectors and have selectively targeted checkpoint inhibitors also in the field of haplo-HSCT, where NK cells and γδ T lymphocytes early provide protection against cancers. Although helper ILCs could theoretically play a key role against tumors, the investigations of their clinical and functional impacts following HSCT are still in their infancy and must be deeper exploited. Our challenges and clinical perspectives over the next decade rely on our ability to give answers to the several important biological questions we still have on these matters.

## Author Contributions

EZ, MC, CD, and DM wrote the manuscript and approved the final version.

### Conflict of Interest

The authors declare that the research was conducted in the absence of any commercial or financial relationships that could be construed as a potential conflict of interest.
